# Bis[5-chloro-2-(phenyl­diazenyl-κ*N* ^2^)pyridine-κ*N*]bis­(thio­cyanato-κ*N*)iron(II)

**DOI:** 10.1107/S1600536812014286

**Published:** 2012-04-13

**Authors:** Luksamee Vittaya, Nararak Leesakul, Chaveng Pakawatchai, Saowanit Saithong, Kanidtha Hansongnern

**Affiliations:** a Faculty of Science and Fisheries Technology, Rajamangala University of Technology Srivijaya, Sikao, Trang 92150, Thailand; b Department of Chemistry and Center for Innovation in Chemistry, Faculty of Science, Prince of Songkla University, Hat Yai, Songkhla 90112, Thailand

## Abstract

In the title complex, [Fe(NCS)_2_(C_11_H_8_ClN_3_)_2_], the Fe^II^ atom is coordinated by two N atoms from the thio­cyanate ligands and four N atoms from two chelating 5-chloro-2-(phenyl­diazen­yl)pyridine ligands, generating a fairly regular FeN_6_ octa­hedral coordination geometry. The thio­cyanate ions are in a *cis* disposition and the pyridine N atoms are in a *trans* orientation. In the crystal, a short inter­molecular Cl⋯S contact [3.366 (3) Å] is observed.

## Related literature

For background to diazenyl complexes, see: Krause & Krause (1980 ▶); Santra *et al.* (1999 ▶); Hotze, Caspers *et al.* (2004 ▶); Hotze, Kooijman *et al.* (2004 ▶). For applications of diazenyl compounds, see: Erkkila *et al.* (1999 ▶); Wong & Giandomenico (1999 ▶); Velder *et al.* (2000 ▶); Barf & Sheldon (1995 ▶). For structures of related diazenyl­imine complexes, see: Hansongnern *et al.* (2008 ▶); Ray *et al.* (2005 ▶); Senapoti *et al.* (2002) ▶. For background to diazenyl complexes, see: Byabartta *et al.* (2001 ▶).
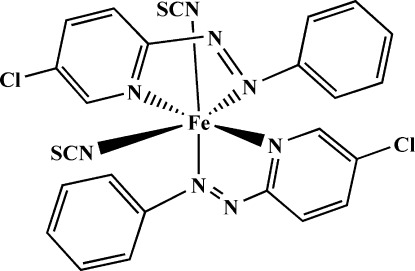


## Experimental

### 

#### Crystal data

[Fe(NCS)_2_(C_11_H_8_ClN_3_)_2_]
*M* *_r_* = 607.34
Monoclinic, 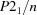

*a* = 9.5151 (3) Å
*b* = 23.7391 (9) Å
*c* = 12.1550 (4) Å
β = 96.209 (1)°
*V* = 2729.46 (16) Å^3^
*Z* = 4
Mo *K*α radiation
μ = 0.93 mm^−1^
*T* = 293 K
0.34 × 0.17 × 0.09 mm

#### Data collection

Bruker SMART APEX CCD diffractometer
Absorption correction: multi-scan (*SADABS*; Bruker, 2003 ▶) *T*_min_ = 0.916, *T*_max_ = 1.000
29379 measured reflections
4809 independent reflections
4189 reflections with *I* > 2σ(*I*)
*R*_int_ = 0.027

#### Refinement

*R*[*F* ^2^ > 2σ(*F* ^2^)] = 0.043
*wR*(*F* ^2^) = 0.110
*S* = 1.08
4809 reflections
334 parameters
H-atom parameters constrained
Δρ_max_ = 0.95 e Å^−3^
Δρ_min_ = −0.75 e Å^−3^

### 

Data collection: *SMART* (Bruker, 1998 ▶); cell refinement: *SAINT* (Bruker, 2003 ▶); data reduction: *SAINT*; program(s) used to solve structure: *SHELXS97* (Sheldrick, 2008 ▶); program(s) used to refine structure: *SHELXL97* (Sheldrick, 2008 ▶); molecular graphics: *Mercury* (Macrae *et al.*, 2008 ▶); software used to prepare material for publication: *SHELXTL* (Sheldrick, 2008 ▶), *WinGX* (Farrugia, 1999 ▶) and *publCIF* (Westrip, 2010 ▶).

## Supplementary Material

Crystal structure: contains datablock(s) I, global. DOI: 10.1107/S1600536812014286/hb6716sup1.cif

Structure factors: contains datablock(s) I. DOI: 10.1107/S1600536812014286/hb6716Isup2.hkl

Additional supplementary materials:  crystallographic information; 3D view; checkCIF report

## Figures and Tables

**Table 1:** Selected bond lengths (Å)

Fe1—N3	1.900 (2)
Fe1—N6	1.917 (2)
Fe1—N4	1.936 (2)
Fe1—N7	1.941 (2)
Fe1—N1	1.945 (2)
Fe1—N8	1.952 (2)

**Fig. 1.**
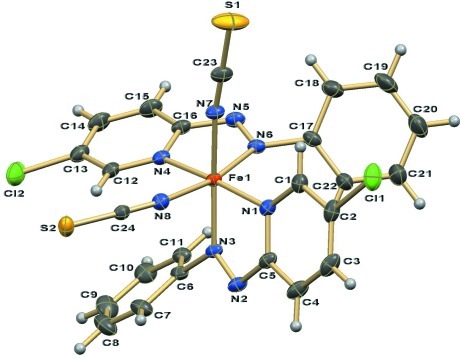
Molecular structure of the title compound with displacement ellipsoids plotted at the 50% probability level.

**Fig. 2.**
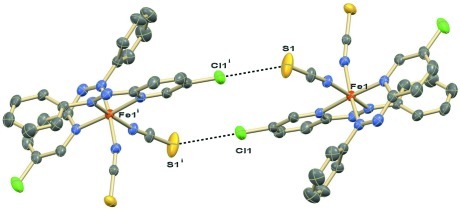
The short contact interactions of [Fe(Clazpy)2(NCS)2] .

## supplementary crystallographic information

### Comment

Azoimine (—N=N—C=N—) compounds are famously used as effective ligands in synthesization of transition metal complexes owing of their strong π-acidity (Krause and Krause 1980; Santra *et al.*, 1999; Hotze, Caspers *et al.*, 2004), stability and various applications like DNA probing (Erkkila *et al*., 1999), chemotherapeutic drugs (Wong *et al.*, 1999), anticancer activity (Hotze, Kooijman *et al.*, 2004) and powerful catalysts in epoxidation reaction of olefin to give epoxide (Barf and Sheldon 1995). The diazenylimine complexes are mostly found in octahedral geometry with d^6^ metal ions (Hansongnern *et al.*, 2008; Ray *et al.*, 2005; Senapoti *et al*., 2002).

Herein, we report the synthesis and crystal structure of a new Fe(II) complex with 5-chloro-2-(phenyldiazenyl) pyridine, (C_11_H_8_N_3_Cl_1_: Clazpy), an azoimine ligand. Regarding to the title compound, the molecular structure of [Fe(Clazpy)_2_(NCS)_2_] is a distorted octahedral complex (Scheme 1 and Fig.1). The chelating coordination is observed by two N atoms from pyridine rings [Fe(1)—N(1) = 1.945 (2) Å and Fe(1)—N(4) = 1.936 (2) Å] and other two N atoms from diazenyl moiety [Fe(1)—N(3) = 1.900 (2) Å and Fe(1)—N(6) = 1.917 (2) Å in *trans* arrangement while two N atoms of both thiocyanato ligands are in *cis* geometry [Fe(1)—N(7) = 1.941 (2) Å, Fe(1)—N(8) = 1.952 (2) Å]. The dihedral angles between pyridine and phenyl rings of both Clazpy ligands are similar, with 53.83 (12)° and 52.53 (10)°. The bond lengths of Fe—N(NCS) (1.941 (2) Å and 1.952 (2) Å) are longer than that reported in the related complex, [Fe(MeaaiEt)_2_(NCS)_2_]; MeaaiEt = 1-ethyl-2(*p*-tolyldiazenyl)imiddiazenylle (Ray *et al.*, 2005). The average Fe—N(py) and Fe—N(diazenyl) distances (1.9085 Å and 1.9405 Å) in [Fe(Clazpy)_2_(NCS)_2_] is shorter than that observed in [Fe(MeaaiEt)_2_(NCS)_2_], Fe—*N*(imiddiazenylle) = 2.103 (2) Å and Fe—N(diazenyl) = 2.371 (2) Å, supporting the strong σ-donor and π-acceptor property of Clazpy. The better π-back bonding from d^6^-Fe(II) to π* orbital of the Clazpy ligand makes slightly N=N distance to be longer comparison with MeaaiEt owing to the decreasing of N=N bond order, thus, the strength of the diazenyl bond decreases. All N(py)—Fe—N(py) and N(diazenyl)—Fe—NCS bond angles deviate from 180°, especially for N(6)—Fe(1)—N(8) = 168.44 (10)° during to the bite angle from the two Clazpy ligands. The torsion angles of pyridine-diazenyl-phenyl atoms, C(5)—N(2)—N(3)—C(6) and C(16)—N(5)—N(6)—C(17), are -176.4 (2) and -178.6 (2)°, respectively. In addition, the short contact between Cl(1) of clazpy and S(1) of isothiocyanato ligand are observed (Cl(1)···S(1) = 3.366 (3) Å) between two adjacent molecules.

### Experimental

Methanolic solution (30 ml) of 5-chloro-2-(phenyldiazenyl)pyridine (Clazpy) (0.11 g, 0.50 mmol), FeSO_4_.7H_2_O (0.07 g, 0.25 mmol) and ammonium thiocynate (0.04 g, 0.53 mmol) was refluxed for 3 h. The filtrate was standing for overnight at room temperature. The green solids were precipitated and collected by filtration, washed it with methanol/water (1:1 *v*/*v*), and dried *in vacuo* for a day. The green solids were recrystallized in the mixture of CH_2_Cl_2_ and MeOH (1:2). The green crystals were obtained (yield 80%, 0.12 g). Anal. Calcd for FeC_24_H_16_N_8_ S_2_Cl_2_: C, 47.47; H, 2.66; N, 18.45; S, 10.56. Found: C, 47.07; H, 2.56; N, 17.98; S, 10.39.

### Refinement

All hydrogen atoms were constrained, C—H = 0.93 Å with *U*_iso_(H) = 1.2*U*_eq_(C) for C-*sp*^2^ atoms of pyridine and phenyl rings.

### Crystal data

**Table:**

[Fe(NCS)_2_(C_11_H_8_ClN_3_)_2_]	*F*(000) = 1232
*M**_r_* = 607.34	*D*_x_ = 1.478 Mg m^−^^3^
Monoclinic, *P*2_1_/*n*	Mo *K*α radiation, λ = 0.71073 Å
Hall symbol: -P 2yn	Cell parameters from 7953 reflections
*a* = 9.5151 (3) Å	θ = 2.3–25.4°
*b* = 23.7391 (9) Å	µ = 0.93 mm^−^^1^
*c* = 12.1550 (4) Å	*T* = 293 K
β = 96.209 (1)°	Block, red-brown
*V* = 2729.46 (16) Å^3^	0.34 × 0.17 × 0.09 mm
*Z* = 4	

### Data collection

**Table:**

Bruker SMART APEX CCD diffractometer	4809 independent reflections
Radiation source: fine-focus sealed tube	4189 reflections with *I* > 2σ(*I*)
Graphite monochromator	*R*_int_ = 0.027
Frames, each covering 0.3 ° in ω scans	θ_max_ = 25.0°, θ_min_ = 1.7°
Absorption correction: multi-scan (*SADABS*; Bruker, 2003)	*h* = −11→11
*T*_min_ = 0.916, *T*_max_ = 1.000	*k* = −28→28
29379 measured reflections	*l* = −14→14

### Refinement

**Table:**

Refinement on *F*^2^	Primary atom site location: structure-invariant direct methods
Least-squares matrix: full	Secondary atom site location: difference Fourier map
*R*[*F*^2^ > 2σ(*F*^2^)] = 0.043	Hydrogen site location: inferred from neighbouring sites
*wR*(*F*^2^) = 0.110	H-atom parameters constrained
*S* = 1.08	*w* = 1/[σ^2^(*F*_o_^2^) + (0.0511*P*)^2^ + 2.004*P*] where *P* = (*F*_o_^2^ + 2*F*_c_^2^)/3
4809 reflections	(Δ/σ)_max_ = 0.001
334 parameters	Δρ_max_ = 0.95 e Å^−^^3^
0 restraints	Δρ_min_ = −0.75 e Å^−^^3^

### Special details

**Table:**

Geometry. All e.s.d.'s (except the e.s.d. in the dihedral angle between two l.s. planes) are estimated using the full covariance matrix. The cell e.s.d.'s are taken into account individually in the estimation of e.s.d.'s in distances, angles and torsion angles; correlations between e.s.d.'s in cell parameters are only used when they are defined by crystal symmetry. An approximate (isotropic) treatment of cell e.s.d.'s is used for estimating e.s.d.'s involving l.s. planes.
Refinement. Refinement of *F*^2^ against all reflections. The weighted *R*-factor *wR* and goodness of fit *S* are based on *F*^2^, conventional *R*-factors *R* are based on *F*, with *F* set to zero for negative *F*^2^. The threshold expression of *F*^2^ > 2*σ*(*F*^2^) is used only for calculating *R*-factors(*gt*) *etc*. and is not relevant to the choice of reflections for refinement. *R*-factors based on *F*^2^ are statistically about twice as large as those based on *F*, and *R*- factors based on al data will be even larger.

### Fractional atomic coordinates and isotropic or equivalent isotropic displacement parameters (Å^2^)

**Table:**

	*x*	*y*	*z*	*U*_iso_*/*U*_eq_	
Fe1	0.26710 (4)	0.199109 (16)	0.72956 (3)	0.03803 (13)	
N1	0.4302 (2)	0.15150 (10)	0.77250 (18)	0.0439 (5)	
N2	0.4637 (2)	0.22105 (11)	0.90825 (19)	0.0513 (6)	
N3	0.3387 (2)	0.23370 (10)	0.86513 (17)	0.0415 (5)	
N4	0.1056 (2)	0.24866 (10)	0.70140 (17)	0.0432 (5)	
N5	−0.0086 (2)	0.16663 (12)	0.7497 (2)	0.0536 (6)	
N6	0.1197 (2)	0.15144 (10)	0.77242 (18)	0.0451 (5)	
N7	0.2284 (2)	0.16225 (10)	0.58695 (19)	0.0490 (6)	
N8	0.3889 (2)	0.25196 (11)	0.66138 (19)	0.0470 (5)	
S2	0.49476 (8)	0.34626 (3)	0.56230 (8)	0.0626 (2)	
S1	0.20586 (13)	0.09807 (7)	0.39640 (12)	0.1316 (7)	
C1	0.4725 (3)	0.10415 (13)	0.7267 (3)	0.0522 (7)	
H1	0.4145	0.0873	0.6694	0.063*	
C2	0.6012 (3)	0.07977 (14)	0.7631 (3)	0.0613 (9)	
C3	0.6913 (3)	0.10504 (17)	0.8452 (3)	0.0713 (10)	
H3	0.7798	0.0898	0.8675	0.086*	
C4	0.6470 (3)	0.15305 (17)	0.8927 (3)	0.0667 (9)	
H4	0.7047	0.1709	0.9488	0.080*	
C5	0.5151 (3)	0.17485 (14)	0.8567 (2)	0.0494 (7)	
C6	0.2817 (3)	0.28345 (12)	0.9103 (2)	0.0458 (6)	
C7	0.3583 (4)	0.33294 (15)	0.9139 (3)	0.0676 (9)	
H7	0.4501	0.3333	0.8943	0.081*	
C8	0.2971 (5)	0.38131 (17)	0.9467 (4)	0.0913 (13)	
H8	0.3475	0.4149	0.9496	0.110*	
C9	0.1606 (5)	0.38048 (18)	0.9755 (3)	0.0905 (13)	
H9	0.1187	0.4137	0.9958	0.109*	
C10	0.0864 (4)	0.33076 (17)	0.9743 (3)	0.0717 (10)	
H10	−0.0043	0.3303	0.9961	0.086*	
C11	0.1460 (3)	0.28201 (14)	0.9411 (2)	0.0520 (7)	
H11	0.0958	0.2483	0.9392	0.062*	
C12	0.1023 (3)	0.30337 (13)	0.6782 (2)	0.0496 (7)	
H12	0.1861	0.3220	0.6685	0.059*	
C13	−0.0229 (3)	0.33307 (15)	0.6681 (3)	0.0611 (8)	
C14	−0.1487 (4)	0.30664 (18)	0.6787 (3)	0.0751 (11)	
H14	−0.2334	0.3265	0.6711	0.090*	
C15	−0.1460 (3)	0.25012 (18)	0.7007 (3)	0.0711 (10)	
H15	−0.2297	0.2307	0.7069	0.085*	
C16	−0.0178 (3)	0.22207 (14)	0.7138 (2)	0.0519 (7)	
C17	0.1347 (3)	0.09386 (12)	0.8076 (2)	0.0503 (7)	
C18	0.0563 (4)	0.05231 (16)	0.7497 (3)	0.0752 (10)	
H18	−0.0066	0.0612	0.6882	0.090*	
C19	0.0737 (5)	−0.00269 (18)	0.7856 (4)	0.0987 (15)	
H19	0.0232	−0.0313	0.7470	0.118*	
C20	0.1640 (5)	−0.01550 (17)	0.8771 (5)	0.0998 (15)	
H20	0.1738	−0.0527	0.9007	0.120*	
C21	0.2400 (4)	0.02575 (16)	0.9341 (4)	0.0832 (11)	
H21	0.3010	0.0167	0.9966	0.100*	
C22	0.2266 (3)	0.08071 (13)	0.8994 (3)	0.0580 (8)	
H22	0.2792	0.1089	0.9377	0.070*	
C23	0.2177 (3)	0.13531 (15)	0.5084 (3)	0.0592 (8)	
C24	0.4349 (3)	0.29088 (12)	0.6208 (2)	0.0423 (6)	
Cl1	0.64768 (11)	0.01811 (4)	0.70172 (10)	0.0874 (3)	
Cl2	−0.01594 (12)	0.40406 (4)	0.64356 (10)	0.0929 (3)	

### Atomic displacement parameters (Å^2^)

**Table:**

	*U*^11^	*U*^22^	*U*^33^	*U*^12^	*U*^13^	*U*^23^
Fe1	0.0315 (2)	0.0475 (2)	0.0347 (2)	0.00046 (15)	0.00200 (14)	−0.00205 (15)
N1	0.0344 (11)	0.0541 (14)	0.0438 (12)	0.0022 (10)	0.0072 (9)	0.0068 (10)
N2	0.0360 (12)	0.0753 (17)	0.0416 (13)	−0.0022 (11)	−0.0004 (10)	0.0006 (12)
N3	0.0342 (11)	0.0558 (14)	0.0344 (11)	−0.0058 (10)	0.0034 (9)	0.0012 (10)
N4	0.0372 (12)	0.0567 (14)	0.0348 (11)	0.0067 (10)	−0.0001 (9)	−0.0054 (10)
N5	0.0361 (13)	0.0714 (17)	0.0526 (14)	−0.0047 (11)	0.0020 (10)	−0.0058 (12)
N6	0.0369 (12)	0.0562 (14)	0.0422 (12)	−0.0057 (10)	0.0040 (9)	−0.0072 (10)
N7	0.0446 (13)	0.0613 (15)	0.0404 (13)	0.0026 (11)	0.0017 (10)	−0.0108 (11)
N8	0.0423 (13)	0.0547 (14)	0.0447 (13)	0.0013 (11)	0.0084 (10)	0.0003 (11)
S2	0.0505 (4)	0.0526 (5)	0.0840 (6)	0.0021 (3)	0.0047 (4)	0.0187 (4)
S1	0.0840 (8)	0.1876 (15)	0.1193 (10)	0.0205 (8)	−0.0075 (7)	−0.1068 (11)
C1	0.0482 (16)	0.0534 (17)	0.0567 (18)	0.0057 (13)	0.0130 (13)	0.0084 (14)
C2	0.0548 (18)	0.066 (2)	0.067 (2)	0.0172 (16)	0.0222 (16)	0.0224 (16)
C3	0.0431 (17)	0.105 (3)	0.067 (2)	0.0237 (18)	0.0115 (16)	0.025 (2)
C4	0.0398 (16)	0.104 (3)	0.0553 (19)	0.0097 (17)	−0.0004 (14)	0.0079 (18)
C5	0.0355 (14)	0.0702 (19)	0.0423 (15)	−0.0004 (13)	0.0026 (12)	0.0084 (14)
C6	0.0485 (16)	0.0556 (17)	0.0330 (13)	−0.0068 (13)	0.0030 (11)	−0.0059 (12)
C7	0.066 (2)	0.071 (2)	0.067 (2)	−0.0203 (18)	0.0130 (17)	−0.0172 (17)
C8	0.122 (4)	0.066 (2)	0.088 (3)	−0.027 (2)	0.024 (3)	−0.024 (2)
C9	0.127 (4)	0.070 (3)	0.079 (3)	0.012 (3)	0.031 (3)	−0.023 (2)
C10	0.076 (2)	0.087 (3)	0.056 (2)	0.010 (2)	0.0224 (17)	−0.0098 (18)
C11	0.0513 (17)	0.0639 (19)	0.0417 (15)	−0.0032 (14)	0.0093 (12)	−0.0030 (13)
C12	0.0478 (16)	0.0626 (19)	0.0369 (14)	0.0100 (14)	−0.0019 (12)	−0.0028 (13)
C13	0.060 (2)	0.072 (2)	0.0484 (17)	0.0227 (17)	−0.0063 (14)	−0.0040 (15)
C14	0.048 (2)	0.102 (3)	0.072 (2)	0.0312 (19)	−0.0086 (16)	−0.005 (2)
C15	0.0358 (16)	0.100 (3)	0.075 (2)	0.0076 (17)	−0.0038 (15)	−0.003 (2)
C16	0.0358 (15)	0.073 (2)	0.0459 (16)	0.0016 (14)	−0.0026 (12)	−0.0059 (14)
C17	0.0450 (15)	0.0504 (17)	0.0568 (17)	−0.0108 (13)	0.0118 (13)	−0.0095 (13)
C18	0.069 (2)	0.071 (2)	0.083 (2)	−0.0176 (18)	−0.0039 (19)	−0.0160 (19)
C19	0.097 (3)	0.064 (3)	0.133 (4)	−0.033 (2)	0.001 (3)	−0.022 (3)
C20	0.101 (3)	0.055 (2)	0.141 (4)	−0.019 (2)	0.001 (3)	0.006 (3)
C21	0.091 (3)	0.061 (2)	0.095 (3)	−0.014 (2)	−0.003 (2)	0.016 (2)
C22	0.0599 (19)	0.0500 (18)	0.0631 (19)	−0.0108 (14)	0.0020 (15)	0.0015 (14)
C23	0.0355 (15)	0.078 (2)	0.063 (2)	0.0087 (14)	−0.0004 (13)	−0.0181 (17)
C24	0.0346 (13)	0.0487 (16)	0.0428 (14)	0.0077 (12)	0.0004 (11)	−0.0032 (12)
Cl1	0.0880 (7)	0.0692 (6)	0.1091 (8)	0.0340 (5)	0.0298 (6)	0.0175 (5)
Cl2	0.1028 (8)	0.0715 (6)	0.1004 (8)	0.0383 (6)	−0.0082 (6)	0.0028 (5)

### Geometric parameters (Å, º)

**Table:**

Fe1—N3	1.900 (2)	C6—C11	1.384 (4)
Fe1—N6	1.917 (2)	C7—C8	1.366 (5)
Fe1—N4	1.936 (2)	C7—H7	0.9300
Fe1—N7	1.941 (2)	C8—C9	1.381 (6)
Fe1—N1	1.945 (2)	C8—H8	0.9300
Fe1—N8	1.952 (2)	C9—C10	1.375 (6)
N1—C1	1.336 (4)	C9—H9	0.9300
N1—C5	1.352 (4)	C10—C11	1.369 (5)
N2—N3	1.282 (3)	C10—H10	0.9300
N2—C5	1.379 (4)	C11—H11	0.9300
N3—C6	1.433 (4)	C12—C13	1.378 (4)
N4—C12	1.329 (4)	C12—H12	0.9300
N4—C16	1.356 (4)	C13—C14	1.370 (5)
N5—N6	1.274 (3)	C13—Cl2	1.714 (4)
N5—C16	1.386 (4)	C14—C15	1.368 (5)
N6—C17	1.435 (4)	C14—H14	0.9300
N7—C23	1.144 (4)	C15—C16	1.384 (4)
N8—C24	1.156 (4)	C15—H15	0.9300
S2—C24	1.626 (3)	C17—C22	1.378 (4)
S1—C23	1.617 (3)	C17—C18	1.382 (4)
C1—C2	1.383 (4)	C18—C19	1.381 (6)
C1—H1	0.9300	C18—H18	0.9300
C2—C3	1.380 (5)	C19—C20	1.364 (6)
C2—Cl1	1.722 (4)	C19—H19	0.9300
C3—C4	1.365 (5)	C20—C21	1.361 (6)
C3—H3	0.9300	C20—H20	0.9300
C4—C5	1.384 (4)	C21—C22	1.373 (5)
C4—H4	0.9300	C21—H21	0.9300
C6—C7	1.380 (4)	C22—H22	0.9300
			
N3—Fe1—N6	102.92 (9)	C8—C7—H7	120.5
N3—Fe1—N4	95.39 (9)	C6—C7—H7	120.5
N6—Fe1—N4	79.44 (10)	C7—C8—C9	120.2 (4)
N3—Fe1—N7	169.99 (9)	C7—C8—H8	119.9
N6—Fe1—N7	84.43 (10)	C9—C8—H8	119.9
N4—Fe1—N7	92.62 (9)	C10—C9—C8	120.4 (4)
N3—Fe1—N1	79.49 (10)	C10—C9—H9	119.8
N6—Fe1—N1	99.78 (10)	C8—C9—H9	119.8
N4—Fe1—N1	174.56 (9)	C11—C10—C9	120.0 (3)
N7—Fe1—N1	92.66 (10)	C11—C10—H10	120.0
N3—Fe1—N8	85.23 (9)	C9—C10—H10	120.0
N6—Fe1—N8	168.44 (10)	C10—C11—C6	119.2 (3)
N4—Fe1—N8	91.75 (10)	C10—C11—H11	120.4
N7—Fe1—N8	88.54 (10)	C6—C11—H11	120.4
N1—Fe1—N8	89.68 (10)	N4—C12—C13	121.3 (3)
C1—N1—C5	118.6 (2)	N4—C12—H12	119.3
C1—N1—Fe1	130.1 (2)	C13—C12—H12	119.3
C5—N1—Fe1	111.03 (19)	C14—C13—C12	120.9 (3)
N3—N2—C5	111.1 (2)	C14—C13—Cl2	121.1 (3)
N2—N3—C6	114.1 (2)	C12—C13—Cl2	118.0 (3)
N2—N3—Fe1	118.86 (18)	C15—C14—C13	118.0 (3)
C6—N3—Fe1	125.00 (17)	C15—C14—H14	121.0
C12—N4—C16	118.5 (2)	C13—C14—H14	121.0
C12—N4—Fe1	129.2 (2)	C14—C15—C16	119.5 (3)
C16—N4—Fe1	112.2 (2)	C14—C15—H15	120.3
N6—N5—C16	111.3 (2)	C16—C15—H15	120.3
N5—N6—C17	113.3 (2)	N4—C16—C15	121.8 (3)
N5—N6—Fe1	119.1 (2)	N4—C16—N5	116.8 (2)
C17—N6—Fe1	126.25 (18)	C15—C16—N5	121.2 (3)
C23—N7—Fe1	171.1 (3)	C22—C17—C18	120.7 (3)
C24—N8—Fe1	164.8 (2)	C22—C17—N6	119.4 (2)
N1—C1—C2	121.0 (3)	C18—C17—N6	119.9 (3)
N1—C1—H1	119.5	C19—C18—C17	118.3 (4)
C2—C1—H1	119.5	C19—C18—H18	120.8
C3—C2—C1	120.6 (3)	C17—C18—H18	120.8
C3—C2—Cl1	120.9 (3)	C20—C19—C18	120.8 (4)
C1—C2—Cl1	118.5 (3)	C20—C19—H19	119.6
C4—C3—C2	118.2 (3)	C18—C19—H19	119.6
C4—C3—H3	120.9	C21—C20—C19	120.5 (4)
C2—C3—H3	120.9	C21—C20—H20	119.7
C3—C4—C5	119.3 (3)	C19—C20—H20	119.7
C3—C4—H4	120.4	C20—C21—C22	120.0 (4)
C5—C4—H4	120.4	C20—C21—H21	120.0
N1—C5—N2	117.2 (2)	C22—C21—H21	120.0
N1—C5—C4	122.2 (3)	C21—C22—C17	119.6 (3)
N2—C5—C4	120.5 (3)	C21—C22—H22	120.2
C7—C6—C11	121.1 (3)	C17—C22—H22	120.2
C7—C6—N3	119.6 (3)	N7—C23—S1	178.5 (3)
C11—C6—N3	119.1 (3)	N8—C24—S2	178.2 (2)
C8—C7—C6	119.0 (3)		
			
N3—Fe1—N1—C1	173.6 (3)	Cl1—C2—C3—C4	−178.3 (3)
N6—Fe1—N1—C1	72.2 (2)	C2—C3—C4—C5	−0.6 (5)
N7—Fe1—N1—C1	−12.7 (2)	C1—N1—C5—N2	−174.8 (2)
N8—Fe1—N1—C1	−101.2 (2)	Fe1—N1—C5—N2	10.3 (3)
N3—Fe1—N1—C5	−12.28 (18)	C1—N1—C5—C4	3.5 (4)
N6—Fe1—N1—C5	−113.74 (19)	Fe1—N1—C5—C4	−171.4 (2)
N7—Fe1—N1—C5	161.45 (19)	N3—N2—C5—N1	0.3 (4)
N8—Fe1—N1—C5	72.93 (19)	N3—N2—C5—C4	−178.0 (3)
C5—N2—N3—C6	−176.4 (2)	C3—C4—C5—N1	−2.7 (5)
C5—N2—N3—Fe1	−11.8 (3)	C3—C4—C5—N2	175.6 (3)
N6—Fe1—N3—N2	111.7 (2)	N2—N3—C6—C7	53.0 (4)
N4—Fe1—N3—N2	−167.9 (2)	Fe1—N3—C6—C7	−110.6 (3)
N7—Fe1—N3—N2	−24.9 (7)	N2—N3—C6—C11	−132.3 (3)
N1—Fe1—N3—N2	14.0 (2)	Fe1—N3—C6—C11	64.1 (3)
N8—Fe1—N3—N2	−76.6 (2)	C11—C6—C7—C8	−1.1 (5)
N6—Fe1—N3—C6	−85.4 (2)	N3—C6—C7—C8	173.4 (3)
N4—Fe1—N3—C6	−5.0 (2)	C6—C7—C8—C9	−0.1 (6)
N7—Fe1—N3—C6	138.0 (5)	C7—C8—C9—C10	1.7 (7)
N1—Fe1—N3—C6	176.8 (2)	C8—C9—C10—C11	−2.1 (6)
N8—Fe1—N3—C6	86.3 (2)	C9—C10—C11—C6	0.8 (5)
N3—Fe1—N4—C12	67.2 (2)	C7—C6—C11—C10	0.8 (5)
N6—Fe1—N4—C12	169.3 (2)	N3—C6—C11—C10	−173.8 (3)
N7—Fe1—N4—C12	−106.8 (2)	C16—N4—C12—C13	0.8 (4)
N8—Fe1—N4—C12	−18.2 (2)	Fe1—N4—C12—C13	−175.3 (2)
N3—Fe1—N4—C16	−109.20 (19)	N4—C12—C13—C14	−1.9 (5)
N6—Fe1—N4—C16	−7.03 (18)	N4—C12—C13—Cl2	177.4 (2)
N7—Fe1—N4—C16	76.81 (19)	C12—C13—C14—C15	0.7 (5)
N8—Fe1—N4—C16	165.42 (19)	Cl2—C13—C14—C15	−178.5 (3)
C16—N5—N6—C17	−178.6 (2)	C13—C14—C15—C16	1.3 (5)
C16—N5—N6—Fe1	−11.1 (3)	C12—N4—C16—C15	1.3 (4)
N3—Fe1—N6—N5	103.7 (2)	Fe1—N4—C16—C15	178.1 (2)
N4—Fe1—N6—N5	10.6 (2)	C12—N4—C16—N5	−173.2 (2)
N7—Fe1—N6—N5	−83.1 (2)	Fe1—N4—C16—N5	3.6 (3)
N1—Fe1—N6—N5	−174.9 (2)	C14—C15—C16—N4	−2.4 (5)
N8—Fe1—N6—N5	−30.3 (6)	C14—C15—C16—N5	171.9 (3)
N3—Fe1—N6—C17	−90.5 (2)	N6—N5—C16—N4	4.5 (4)
N4—Fe1—N6—C17	176.3 (2)	N6—N5—C16—C15	−170.0 (3)
N7—Fe1—N6—C17	82.6 (2)	N5—N6—C17—C22	−133.9 (3)
N1—Fe1—N6—C17	−9.1 (2)	Fe1—N6—C17—C22	59.6 (3)
N8—Fe1—N6—C17	135.4 (5)	N5—N6—C17—C18	45.6 (4)
N3—Fe1—N8—C24	−87.5 (9)	Fe1—N6—C17—C18	−120.9 (3)
N6—Fe1—N8—C24	47.9 (11)	C22—C17—C18—C19	−0.8 (6)
N4—Fe1—N8—C24	7.8 (9)	N6—C17—C18—C19	179.8 (3)
N7—Fe1—N8—C24	100.4 (9)	C17—C18—C19—C20	1.2 (7)
N1—Fe1—N8—C24	−167.0 (9)	C18—C19—C20—C21	−0.7 (8)
C5—N1—C1—C2	−0.9 (4)	C19—C20—C21—C22	−0.3 (8)
Fe1—N1—C1—C2	172.8 (2)	C20—C21—C22—C17	0.8 (6)
N1—C1—C2—C3	−2.3 (5)	C18—C17—C22—C21	−0.2 (5)
N1—C1—C2—Cl1	179.0 (2)	N6—C17—C22—C21	179.2 (3)
C1—C2—C3—C4	3.1 (5)		
